# Priming Melon Defenses with Acibenzolar-*S*-methyl Attenuates Infections by Phylogenetically Distinct Viruses and Diminishes Vector Preferences for Infected Hosts

**DOI:** 10.3390/v12030257

**Published:** 2020-02-26

**Authors:** Jaimie R. Kenney, Marie-Eve Grandmont, Kerry E. Mauck

**Affiliations:** 1 Department of Entomology, University of California-Riverside, Riverside, CA 92521, USA; jkenn009@ucr.edu; 2 Gembloux Agro-Bio Tech, University of Liège, B-5030 Gembloux, Belgium; megrandmont@gmail.com

**Keywords:** plant defense elicitor, plant virus, vector behavior, cucurbit yellow stunting disorder virus (CYSDV), cucumber mosaic virus (CMV), *Bemisia tabaci*, *Aphis gossypii*, virus pathology, electrical penetration graphing, virus manipulation

## Abstract

Plant virus management is mostly achieved through control of insect vectors using insecticides. However, insecticides are only marginally effective for preventing virus transmission. Furthermore, it is well established that symptoms of virus infections often encourage vector visitation to infected hosts, which exacerbates secondary spread. Plant defense elicitors, phytohormone analogs that prime the plant immune system against attack, may be a viable approach for virus control that complements insecticide use by disrupting pathologies that attract vectors. To explore this, we tested the effect of a commercial plant elicitor, acibenzolar-*S*-methyl (ASM), on infection rates, virus titers, and symptom development in melon plants inoculated with one of two virus species, *Cucumber mosaic virus* (CMV) and *Cucurbit yellow stunting disorder virus* (CYSDV). We also conducted behavioral assays to assess the effect of ASM treatment and virus inoculation on vector behavior. For both pathogens, ASM treatment reduced symptom severity and delayed disease progression. For CYSDV, this resulted in the attenuation of symptoms that encourage vector visitation and virion uptake. We did observe slight trade-offs in growth vs. defense following ASM treatment, but these effects did not translate into reduced yields or plant performance in the field. Our results suggest that immunity priming may be a valuable tool for improving management of insect-transmitted plant viruses.

## 1. Introduction

Emerging diseases are defined as infections that have recently increased in recorded incidence or severity within a geographic region [1]. Among emerging infectious diseases of plants, viruses are the number one causal agents [2]. Although it is difficult to measure the exact impact plant viruses have on agricultural production, it is estimated that they are responsible for upwards of USD 30 billion in crop losses each year [3]. Furthermore, the vast majority of characterized plant pathogenic viruses are insect transmitted [4], and climate change and global trade are predicted to alter the distribution of insect vectors in ways that will only increase the number of emerging insect-borne viruses threatening crops in the future [5]. Thus, we urgently need new solutions for plant virus management that are both sustainable and adaptable to multiple virus threats.

Current plant virus management strategies are focused on preventing infection [3,6,7,8,9]. This approach is logical because plant viruses are obligate intracellular parasites and are thus very difficult to eliminate from hosts once established. Several tools have been developed to prevent infections, including clean plant programs that ensure propagation of virus-free tissue across trade zones, cultural management of inoculum sources, and breeding or bioengineering cultivars that are not susceptible to infection. However, developing and implementing these approaches for each new virus threat is not always practical, because they rely on knowledge of virus ecology, drivers of pathology, and host resistance traits, each of which takes years of research to understand. Since most economically damaging plant viruses depend on insect vectors for transmission, growers are often left with no choice but to apply excessive amounts of pesticides to their crops in the hopes of inhibiting virus transmission [9,10,11]. Unfortunately, this practice is expensive, has off-target effects on non-vector species, selects for resistance in pests, and, worst of all, is rarely effective in fully blocking virus transmission [9,11,12,13].

Under scenarios where virus inoculum is abundant due to the presence of reservoir hosts, exposure to viruses is a near certainty even when insecticides are keeping vector populations in a crop below economic injury levels for direct damage. In these situations, boosting plant tolerance to viruses may be a more feasible, adaptable, and sustainable solution relative to time- and labor-intensive solutions, such as breeding new cultivars with resistance traits. Tolerance is defined as the ability to become infected without developing disease. Enhancing tolerance can be an effective control strategy, because most of the negative effects of plant viruses on crop yield and quality can be attributed to the development of symptoms associated with virus-induced pathologies, rather than virus infection itself [14]. In fact, many plant viruses have no known negative impacts on their hosts [15], and many are prevalent but asymptomatic in crop hosts that co-occur geographically alongside crops showing symptoms [16]. In addition to direct impacts on yield, it is also well established that virus symptoms play a role in driving secondary spread from focal infection points [17,18]. Numerous studies across diverse pathosystems demonstrate that virus-induced symptoms often enhance vector attraction to infected hosts and modify feeding behaviors in ways that are conducive to virion uptake, retention, and transmission [18]. Thus, in addition to protecting crop yield and quality, focusing on attenuating viral symptoms may bring the added benefit of decreasing vector attraction to infected plants and significantly reducing secondary transmission rates. 

Tolerance of virus infection is a process that involves the plant immune system. Mechanistic studies have identified signaling molecules (phytohormones) mediating plant perception of, and responses to, beneficial and pathogenic microbes [19]. Based on the structures of these signaling molecules, researchers and industry have developed several synthetic phytohormone analogs that can be used to elicit plant defense responses prior to pathogen attack [20]. One of these so-called plant defense elicitors, acibenzolar-*S*-methyl (ASM, marketed as Actigard by Syngenta), has been labeled for use on crops for the management of several fungal and bacterial plant diseases. More recent studies have demonstrated that ASM also has the potential to protect plants from viral disease. For example, Takeshita et al. [21] found that ASM attenuates symptom development and negative effects of cucurbit chlorotic yellows virus (genus *Crinivirus*, family *Closteroviridae*) on cultivated melon *(Cucumis melo*). Further, Tripathi and Pappu [22] found similar positive effects of ASM on plant tolerance to iris yellow spot virus (genus *Orthotospovirus,* family *Tospoviridae*). While these studies are promising, use of ASM for attenuating virus infection has not been widely tested for efficacy against diverse pathogens affecting the same crop, or for off-target effects on plant growth and productivity. Furthermore, no studies have determined if ASM treatment is effective in disrupting symptoms responsible for increased vector attraction to and feeding on infected hosts.

To address these knowledge gaps, we tested the hypothesis that ASM treatment can enhance *Cucumis melo* resistance or tolerance against two common, but distantly related, virus species (*Cucumber mosaic virus* [CMV, genus *Cucumovirus,* family *Bromoviridae*] and *Cucurbit yellow stunting disorder virus* [CYSDV, genus *Crinivirus,* family *Closteroviridae*]). CMV is a multi-host pathogen that is transmitted at various efficiencies by over 80 different aphid species in a non-persistent manner (i.e., through brief probes of epidermal cells) [23]. Consistent with this transmission mechanism, multiple studies have documented induction of a “pull–push” phenotype in melons and squash infected by CMV; infected plants are initially attractive to aphid vectors via volatile cues, but ultimately prove to be unpalatable once vectors establish contact and perform test probes on epidermal cells [24,25,26,27]. CYSDV is transmitted in a semi-persistent manner by multiple biotypes of *Bemisia tabaci,* which acquire and inoculate the virus during prolonged feeding in phloem sieve-tube elements [28]. In melons, CYSDV induces interveinal chlorosis, resulting in bright yellow leaves at the height of symptom expression, a condition which is hypothesized to be highly attractive to whitefly vectors based on prior studies [29,30]. CYSDV infection also reduces yield size and fruit quality [31,32]. Both CMV and CYSDV are significant threats to melons in regions responsible for the majority of melon production in the U.S. (California and Arizona), the Middle East, and Europe [33,34,35]. For example, in the Southwestern U.S., many growers have ceased planting fall melon crops in desert agriculture due to the presence of CYSDV, resulting in millions of dollars in lost revenue annually [36,37].

In the current study, we explored the effects of ASM pre-treatment on CMV and CYSDV infection rates, symptom progressions, and titers. We also evaluated potential trade-offs in growth vs. defense that may be elicited by ASM application using greenhouse and field studies. Since both pathogens have the potential to influence vector behavior via induced changes in host plant phenotype (symptoms) we further predicted that attenuation of symptoms following ASM pre-treatment would disrupt transmission-conducive vector host-seeking and feeding behaviors (e.g., preference for visiting infected over non-infected hosts). This was tested through a series of behavioral assays with the respective vectors of each virus. Our results suggest that ASM can function as a useful component of integrated disease management programs for CMV and CYSDV, but that careful attention to dose and timing is required to balance trade-offs in growth vs. defense.

## 2. Materials and Methods

### 2.1. Plants, Virus Isolates, and Vectors

All experiments were carried out with melon, *Cucumis melo* var. Gold Express (Syngenta Seeds Inc., Greensboro, NC, USA), germinated in seed flats in a climate-controlled growth chamber (25 ± 1 °C, 65% relative humidity) under a 16 h light/8 h dark photoperiod. One and a half weeks after sowing, seedlings were transplanted to 6 inch diameter × 5.75 inch tall round pots (Kord Regal Standard Pots, Greenhouse Megastore, West Sacramento, CA, USA) and moved to the greenhouse, where natural light and supplemental fluorescent shop lights provided a 16 h light/8 h dark photoperiod. Plants were kept in BugDorm insect rearing tents for the duration of experiments.

The isolate of CYSDV used for all experiments was collected in 2006 from a commercial melon field in Imperial County, CA, and, thus, is known as the Imperial Isolate [16]. It is maintained in live melon in the UC Riverside Insectary and Quarantine facility greenhouse and transmitted to new melon seedlings monthly, using the whitefly vector *B. tabaci*. All experiments involving CMV were performed with isolate KV-PG2, originally collected in 2009 from a field of cultivated squash in Kampsville, IL, USA [25]. Frozen infected melon tissue generated from one cohort of melon plants grown in January 2019 was used for all CMV inoculations in this study.

The *Bemisia tabaci* MEAM1 (formerly biotype B) colony used for this study originated from whiteflies collected in 2006 from cotton at the Maricopa Agricultural Center, Maricopa, AZ, USA [38]. All whiteflies used in this study were sourced from colonies maintained on cowpea (*Vigna unguiculata)* under climate-controlled conditions of 25 ± 1 °C and a 16 h light/8 h dark photoperiod. In addition to the primary bacterial endosymbiont *Portiera aleyrodidarum*, the secondary endosymbiont *Rickettsia* sp. nr. *bellii* is fixed in this laboratory colony.

The *Aphis gossypii* colony used for this study originated from aphids collected from squash near Reedley, CA about a decade ago and reared on melon since then [39]. All aphids used here were reared on melon in the laboratory under climate-controlled conditions of 24 ± 2 °C and supplemental LED lighting providing a 16 h light/8 h dark photoperiod.

### 2.2. ASM Treatment

For all experiments, plants were treated with a foliar spray of 20 mL of 25 ppm (25 mg/L) Actigard (Syngenta, Greensboro, NC, USA) at the one true leaf stage, approximately 1.5 weeks after sowing and one to three days after transplanting and moving to the greenhouse. This dose was selected based on Takeshita et al. [21] and preliminary experiments testing various concentrations for phytotoxic effects. Control plants were treated with a foliar spray of 20 mL of distilled water. After observing a slight reduction in aboveground biomass in plants treated with 20 mL 25 ppm ASM in the greenhouse, we decided to test an additional dosage, 20 mL of 12.5 ppm ASM solution, for our field experiment measuring effects of ASM on melon plant fruit production.

### 2.3. Virus Inoculation

#### 2.3.1. CMV

**Figure 1:**
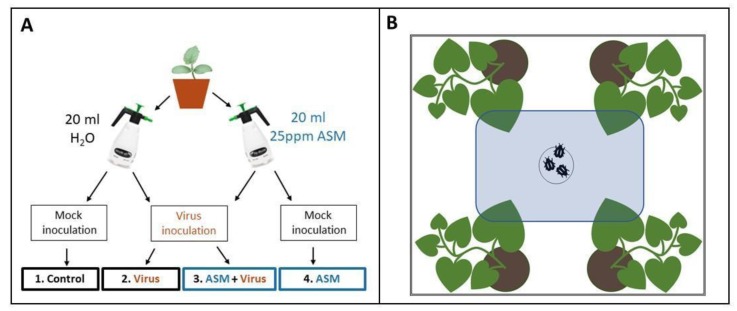
(**A**) Diagram showing the four treatment groups used for vector behavioral assays. At 1.5 weeks old, plants were treated with a foliar spray of either 20 mL of 25 ppm acibenzolar-*S*-methyl (ASM) or 20 mL of water. Three–four days after treatment, they were either inoculated with virus (cucumber mosaic virus [CMV] or cucurbit yellow stunting disorder virus [CYSDV]) or mock-inoculated (with non-viruliferous whiteflies or buffer, respectively). This resulted in four treatment groups: plants that were not treated with ASM or inoculated with virus (1. Control), virus-inoculated plants that were not treated with ASM (2. Virus), plants that were both treated with ASM and inoculated with virus (3. ASM+Virus), and plants that had been treated with ASM but were not inoculated with virus (4. ASM). (**B**) Behavioral assay setup (as seen from above) used to test aphid or whitefly preference between leaves of four treatment groups from **A**. The double black line represents white poster board, the blue box represents a clear, sealed plastic arena with slits for single leaves to pass through the sides. The small black circle represents a hole in the middle of the bottom of the arena where insects were allowed to enter from a small holding area below at the beginning of each test. For CYSDV experiments, approximately 25 whiteflies were released. For CMV experiments, approximately 20 alate aphids were released. Insect positions were recorded at 1, 2, and 24 h after release. Final preferences were quantified based on the total number of insects recovered in each test.

Three days after foliar treatments were applied (Figure 1A), we mechanically inoculated melon seedlings with CMV. Frozen tissue from CMV-infected melon plants was macerated in chilled 0.1M potassium phosphate buffer, mixed with carborundum (−400 mesh particle size, ≥97.5%), and brushed across leaves of non-infected melon seedlings at the 1–2 true leaf stage using a cotton-tipped applicator. The same procedure was used for mock inoculations of control plants with 0.1 M potassium phosphate buffer.

#### 2.3.2. CYSDV

Four days after foliar treatments were applied, we used viruliferous *B. tabaci* to inoculate melon seedlings with CYSDV (Figure 1A). Cohorts of 50–75 *B. tabaci* had previously been placed in clip cages on symptomatic leaves of CYSDV-infected melon plants for a 48 h virus acquisition period. The whiteflies were then released in BugDorms containing melon seedlings at the 1–2 true leaf stage and allowed to feed for 48 h before being removed. For mock inoculations, the same number of non-viruliferous whiteflies were allowed to feed on control plants at the same stage of development and removed after 48 h.

### 2.4. Detection of Viruses and Estimation of Titer

We detected infection and performed a semi-quantitative analysis of virus titer using ELISA (CMV: Alkaline Phosphatase Triple Antibody Sandwich ELISA kit, Agdia, Elkhart, IN, USA; CYSDV: Double Antibody Sandwich ELISA kit, BIOREBA, Kanton Reinach, CH). For CYSDV, we repeated this experiment three times—once with 8 plants per treatment and twice with 6 plants per treatment. However, we did not begin collecting tissue at 3 wpi until the second repetition of the experiment. Thus, we tested a total of 24 CYSDV-inoculated plants (12 ASM treated, 12 non-ASM treated) at 3 wpi and 40 CYSDV-inoculated plants (20 ASM treated, 20 non-ASM treated) at 4 wpi. For CMV, we repeated this experiment three times with 6 plants per treatment each time. However, we did not begin collecting tissue at 1 wpi until the third repetition of the experiment. Therefore, we tested a total of 36 inoculated plants (18 ASM treated, 18 non-ASM treated) at 2 and 3 wpi, but only 12 (6 ASM treated, 6 non-ASM treated) at 1 wpi. Two ⅜ inch diameter leaf disks were taken from each plant, macerated, and mixed with 1 mL of General Extraction Buffer, allowing for standardization of optical density (OD) values as one of two estimates of virus titer (the other being quantitative PCR, described below).

For one repetition of the experiment with CMV (6 plants per treatment) and one repetition of the experiment with CYSDV (6 plants per treatment) we quantified virus titer using quantitative PCR. For CMV, two ⅜ inch diameter leaf disks were taken from one leaf of each plant at 1 wpi, 2 wpi, and 3 wpi and stored at −80 °C until extracting total RNA. For CYSDV, two ⅜ inch diameter leaf disks were taken from one leaf of each plant at 3 wpi and 4 wpi and stored at −80 °C until extracting total RNA. Total RNA was isolated from all samples using RiboZol RNA Extraction Reagent^®^ (AMRESCO, Solon, OH, USA) following the manufacturer’s protocol. Quality and quantity of total RNA was measured using a NanoDrop 2000 UV-Vis spectrophotometer (Thermo Fisher Scientific, Waltham, MA, USA). 1000 ng of RNA from each sample was then reverse transcribed using SuperScript^®^ IV Reverse Transcriptase and random hexamer primers following the manufacturer’s protocol, with one modification to the reaction incubation program: the recommended 10 min incubation at 55 °C was extended to 1 h.

Quantitative PCR (qPCR) was performed using the CFX96 Real-Time PCR Detection System (BioRad, Hercules, CA, USA) and the following thermocycler program: 95 °C for 3 min followed by 40 cycles of 95 °C for 15 s, 60 °C for 30 s, and 72 °C for 30 s. For each qPCR reaction, 10 μL of Luna^®^ Universal qPCR Mastermix (New England BioLabs, Ipswich, MA, USA), 4 μL of UltraPure™ Distilled Water (Invitrogen, Grand Island, NY, USA), and 0.5 μL each of forward and reverse primers were combined with 5 μL of template cDNA. Primers used to amplify cDNA from CYSDV, CMV, and the reference gene *Cucumis melo* β-actin are listed in Table S1. Primer amplification efficiency was calculated by the CFX manager software, with efficiencies ranging between 90% and 110% (Figures S1–S3). Three technical replicates of each sample were performed for each sample with primers targeting the respective virus. Another three technical replicates of each sample with primers targeting the melon reference gene were also performed for subsequent normalization of Ct values and calculation of relative fold virus titer using the delta–delta Ct method [40]. Reactions with virus-free melon negative controls and non-reverse-transcribed controls of each sample were also performed. In order to compare CMV titer both between treatments and across all three timepoints, we calculated the relative fold change in virus titer for each treatment group at each time point relative to the average 1 wpi titer of the plants treated with water before CMV inoculation. To compare CYSDV titer between treatments and across all three timepoints, we calculated the relative fold change in virus titer for each treatment group at each time point relative to the average 3 wpi titer of the plants treated with water before CYSDV inoculation. 

### 2.5. Evaluation of Symptom Severity

We scored symptom severity for each individual leaf larger than 30 mm on a 0–4 scale adapted from Takeshita et al., 2013. We then calculated overall symptom severity for each plant using the formula *D =* [(*d_i_*)/*n*/4] × 100, where *D* is symptom severity of a plant, *d_i_* is symptom rating of *i*th leaf, *n* is the total number of leaves (wider than 3cm) on the plant, and 4 is the rating scale from 1 to 4. For CYSDV, characterized by yellowing symptoms, we used the following: 0 = no symptoms, 1 = slight mottling (tiny light spots visible), 2 ≤ 20% leaf area yellow (but bigger, distinctly yellow spots, rather than tiny light spots), 3 = 21%–50% leaf area yellow, 4 ≥ 50% leaf area yellow. CYSDV symptom severity was evaluated at weeks 3 and 4 post-inoculation, corresponding with typical symptom onset and strong apparency time periods, respectively. This experiment was repeated three times. In the first iteration, we used 8 plants per treatment group. However, due to logistical constraints, we opted to use only 6 plants per treatment for each of the two additional repetitions. This resulted in visual symptom severity scores for a total of 20 plants per treatment group at both the 3 wpi and 4 wpi time points. For CMV, which is characterized by leaf crumpling and mottling, we used the following scoring criteria: 0 = no symptoms (smooth, green leaf), 1= leaf has one “warped” or crumpled spot, but is mostly smooth, 2 = leaf has notable crumpling/wrinkling across whole surface, 3 = whole leaf highly crumpled, but no yellow mottling, 4 = highly crumpled + distinct yellow/light green mottling. CMV symptom severity was evaluated at weeks 1, 2, and 3 post-inoculation, which correspond with typical symptom onset, proliferation (increasing severity), and strong apparency time periods, respectively. This experiment was repeated three times, with 6 plants per treatment group each time. CMV symptom severity was initially evaluated only at 2 and 3 weeks post-inoculation. However, after the first repetition of the experiment, it became clear that an earlier time point would be necessary to capture differences in the initial development of symptoms. Thus, for the following two repetitions of the experiment, symptom severity was also scored at 1 week post-inoculation. This resulted in a total of three repetitions of 6 plants per treatment group (18 total) for visual symptom severity scores at 2 and 3 wpi, and two repetitions of 6 plants per treatment group (12 total) for visual symptom severity at 1 wpi.

### 2.6. Behavioral Assays

We released insect vectors in the middle of the bottom of a clear, rectangular cuboid arena surrounded by white poster board and centered under LED lights (Figure 1B). One plant from each of the four treatment groups was placed randomly outside each corner of the arena, and one leaf of the same age from each plant was pushed through a slit into the arena. The slit was sealed with a strip of white felt to prevent insects from escaping. The number of insects on each of the four leaves was then counted one, two, and twenty-four hours after release. For CYSDV, the assay was repeated with different plants 14 times with approximately 25 whiteflies used for each iteration. Whiteflies had to be chilled for 30s in −20 °C to prevent them from escaping as they were being transferred to the holding area under the arena. This occasionally resulted in one or two fatalities. For CMV, this assay was repeated with different plants 15 times, using approximately 20 alate aphids per iteration. Although winged, they usually did not make any attempt to fly away during transfer to the behavioral assay arena, and, therefore, did not require chilling.

### 2.7. Evaluation of Phytotoxic Effects

To evaluate the side effects of ASM on plant growth, we measured the dry weights of aboveground tissue from plants treated with either 20 mL distilled water or 25 ppm ASM solution at 1.5 weeks of age in the greenhouse, and performed a field experiment to evaluate ASM effects on melon production and quality. In greenhouse experiments, plant shoots were cut off at the soil line, placed in individual paper bags, and dried at 60 °C for one week before weighing. Plants were harvested either at 5 weeks of age (3.5 weeks after ASM application) or at 6 weeks of age (4.5 weeks after ASM application), to evaluate changes in the severity of side effects at different time points in plant phenology post-application. The experiment was repeated twice for plants harvested at 5 weeks of age: the first iteration used 8 plants per treatment group, after which we opted to reduce the number of plants to six per treatment group for the second iteration due to logistical constraints. This resulted in a total of 14 plants per treatment group. The assay was also repeated twice for plants harvested at 6 weeks of age using 6 plants per treatment group each time and, thus, resulting in a total of 12 plants per treatment group. 

For the field experiment, six 1.5 m wide flat-top beds were prepared with furrows in between at the UC Riverside Agricultural Operations facility. Beds were pre-treated with a pre-emergent herbicide (Pre-far) which is typically used prior to melon planting in California. Furrows were treated throughout the season with spot treatments of Roundup (Monsanto, St. Louis, MO, USA) and Reward (Syngenta, Greensboro, NC, USA) to prevent weeds from over-shadowing developing vines. All beds were outfitted with drip tape down the center, which was connected to an automated irrigation system. Melon seedlings (cv. Gold Express) to be used in the field experiment were started in the greenhouse in 36 pot flats. On May 7 2019, we transplanted seedlings to the field when the first true leaves had fully expanded (3.5 weeks post-planting). Beds were well watered prior to planting, and subsequently, all beds were watered for 45 min each day until week 5, at which time the watering was increased to 60 min each day to keep up with transpiration from larger plants. After planting, the beds were divided into plots of four plants each using marking flags, and each plot was assigned randomly to one of three spray treatments: 25ppm ASM, 12.5ppm ASM (half dose), or water (control) (21 plots per treatment). ASM and control treatments were applied seven days after transplanting to allow plants to acclimate. Fertilizer was applied twice during the season through chemigation (Peter’s soluble fertilizer, 20-20-20 NPK, 20bs/acre rate); once shortly after planting, and once during the flowering period (week 6 post-treatment application). Plant size and health assessments were taken at 27 and 39 days post-transplanting. Plant size was rated on a 1–10 scale corresponding to the percent of the bed covered by each plant (1 = 0%–10% covered, 10 = 90%–100% covered) and plant health was rated on a 0–9 scale based on the percent of the plant that was deep green rather than yellow or brown (0 = dead, 1 = 0%–10% green, 9 = 90%–100% green). Melon yields were quantified at the conclusion of the season (three picks over three weeks, selecting melons at full slip during each pick). 

### 2.8. Statistical Analyses

The effect of ASM treatment on virus infection rate was determined using Chi-square tests. Differences in symptom severity, relative titer, and dry weight data between ASM-treated and untreated virus-inoculated plants were determined using independent t-tests or Wilcoxon rank sum tests, depending on whether data were normally distributed or not. All behavioral assay data were analyzed together (for each virus separately) using a generalized linear model with virus treatment, ASM treatment, and their interaction as fixed effects and time point as a random effect (R package ‘lme4′). For fixed effects terms that were significant in the mixed effects models (i.e., virus treatment, ASM treatment, or the interaction), we determined differences within each time point using two-way ANOVAs with interaction effect followed by Tukey’s tests. Melon size, condition, and yield data from our field experiment were analyzed using a one-way ANOVA with ASM treatment as a fixed factor having two levels (25 ppm and 12.5 ppm doses). Statistical differences were considered significant at *p* < 0.05. All statistical analyses were carried out using the statistical program “R” (version 3.5.1) (R Studio Core Team, Boston, MA, USA).

## 3. Results

### 3.1. ASM Effects on Susceptibility to Viruses and Symptom Development

**Figure 2:**
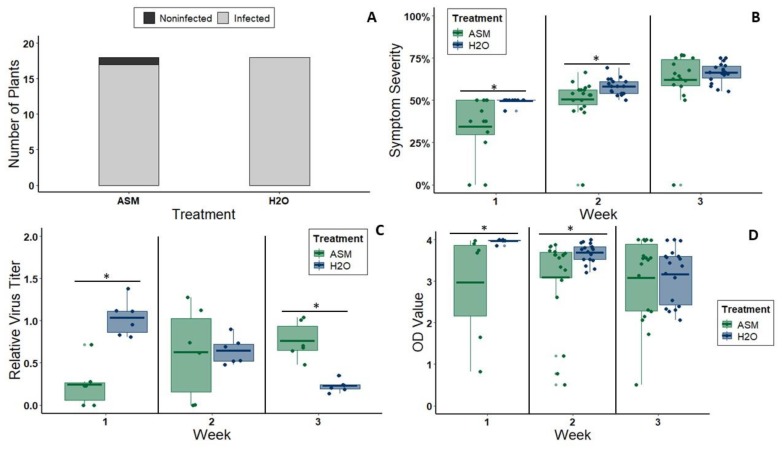
(**A**) Rate of CMV infection as determined by ELISA in plants treated with either 25 ppm ASM (17/18 plants) or water (18/18 plants) and then inoculated with CMV. Differences are not significant by chi-square test. (**B**) Symptom severity of CMV-inoculated melon plants (treated with ASM or water) at three timepoints during each repetition of the same experiment: 1, 2, and 3 wpi. For 1 wpi, *n* = 12 plants per treatment. For 2 and 3 wpi, *n* = 18 plants per treatment. (**C**) Fold change in CMV titer (as determined by qPCR) of tissue from CMV-inoculated melon plants (treated with ASM or water) at three timepoints (1, 2, and 3 wpi) relative to the average 1 wpi titer of the water-treated group. For all three time points *n* = 6 plants per treatment. (**D**) Standardized optical density (OD) values of tissue samples from CMV-inoculated melon plants (treated with ASM or water) tested for CMV infection by ELISA at 1 wpi, 2 wpi, and 3 wpi. For 1 wpi, *n* = 6 plants per treatment (one biological replicate of *n* = 6). For 2 and 3 wpi, *n* = 18 plants per treatment (three biological replicates of *n* = 6). Bars with asterisks denote groups between which there is a significant difference at *p* < 0.05. Dots represent individual data points. The lower and upper edges of boxes represent the first and third quartiles, with the horizontal line inside representing the median value. Whiskers extend to the highest and lowest data points within 1.5× the interquartile range. Outliers beyond this range are represented by additional semi-transparent dots.

For CMV experiments, the first visual symptoms appeared within one week after inoculation. This time frame is consistent with that previously reported for CMV infections in cultivated melons [41]. The infection rate of melon plants treated with 20 mL of 25 ppm ASM solution three days before mechanical CMV inoculation was not different from the infection rate of control plants treated with distilled water (chi-square value = 0, *df* = 1, *p* = 1; Figure 2A). At 1 wpi, ASM-treated plants exhibited significantly reduced symptom severity relative to controls (*n* = 12 per treatment, *W* = 26.5, *p* = 0.003) (Figure 2B), which also corresponded with reduced virus titers (*n* = 6 per treatment, *t* = −5.7245, *df* = 10, *p* = 0.0002) (Figure 2C). Reductions in symptom severity (*n* = 18 per treatment, *W* = 87.5, *p* = 0.019) following ASM treatment persisted at 2 wpi (Figure 2B). Virus titer in 2 wpi ASM-treated plants was equal to that in non-treated plants as determined by qPCR (*n* = 6 per treatment, W = 20, *p* = 0.8102) (Figure 2C) and was slightly lower than non-treated plants according to semi-quantitative ELISA (*n* = 18 per treatment, *W* = 96, *p* = 0.038) (Figure 2D). By 3 wpi, there was no apparent difference in symptom severity between ASM-treated and control plants (*n* = 18 per treatment, *W* = 147, *p* = 0.646) (Figure 2B), but virus titer was significantly higher in the ASM-treated group according to qPCR (*n* = 6 per treatment, *W* = 36, *p* = 0.005075) (Figure 2C). This contrasts with the results of the semi-quantitative ELISA, which showed equivalent titers (*n* = 18 per treatment, W = 161.5, *p* = 1.00) (Figure 2D), suggesting that the ELISA protocol for CMV has an upper limit for measuring differences in titer. 

**Figure 3:**
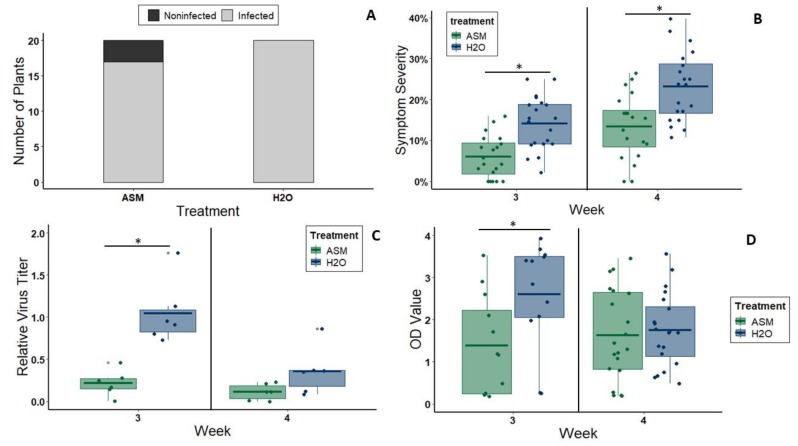
(**A**) Rate of successful CYSDV infection as determined by ELISA in plants treated with either 25 ppm ASM (17/20plants) or water (20/20 plants) and then inoculated with CYSDV via feeding by viruliferous *B. tabaci*. (**B**) Symptom severity of CYSDV-inoculated melon plants (treated with ASM or water) at two timepoints during each repetition of the same experiment: 3 wpi and 4 wpi (*n* = 20 plants per treatment). (**C**) Fold change in CYSDV titer (as determined by qPCR) of tissue from CYSDV-inoculated melon plants (treated with ASM or water) as determined by qPCR at two timepoints (3 and 4 wpi) relative to the average 3 wpi titer of the water-treated group. For both time points *n* = 6 plants per treatment. (**D**) Standardized OD values of tissue samples from CYSDV-inoculated melon plants (treated with ASM or water) tested for CYSDV infection by ELISA at 3 wpi and 4 wpi. For 3 wpi *n* = 12 plants per treatment (two biological replicates of *n* = 6). For 4 wpi *n* = 20 plants per treatment (two biological replicates of *n* = 6 plus one biological replicate of *n* = 8). Bars with asterisks denote groups between which there is a significant difference. Dots represent individual data points. The lower and upper edges of boxes represent the first and third quartiles, with the horizontal line inside representing the median value. Whiskers extend to the highest and lowest data points within 1.5× the interquartile range. Outliers beyond this range are represented by additional semi-transparent dots.

For CYSDV experiments, the first symptoms rarely appeared before three weeks after inoculation. This is consistent with the standard time frame for CYSDV symptom development in cultivated melons [31]. The infection rate of plants treated with 20 mL of 25 ppm ASM solution four days before CYSDV inoculation (via whiteflies) was slightly lower, but not significantly different from the infection rate of control plants treated with distilled water four days before CYSDV inoculation (*chi-square value* = 2.5, *df* = 1, *p* = 0.114; Figure 3A). However, ASM-treated plants exhibited decreased symptom severity relative to controls at both 3 wpi (*n* = 20 per treatment, *t* = −4.39, *df* = 38, *p* < 0.000) and 4 wpi (*n* = 20 per treatment, *t* = −3.80, *df* = 38, *p* = 0.001) (Figure 3B) and reduced virus titers at 3 wpi according to qPCR (*n* = 6 per treatment, *t* = −4.9904, *df* = 10, *p* = 0.0005) (Figure 3C) and semi-quantitative ELISA (*n* = 12 per treatment, W = 32, *p* = 0.023) (Figure 3D). During the fourth week after inoculation, symptom severity increased in both treatments and virus titer in ASM-treated plants was equal to that in non-treated plants according to qPCR (*n* = 6 per treatment, W = 6, *p* = 0.06508) (Figure 3C) and semi-quantitative ELISA (*n* =20 per treatment, t = −0.406, df = 38, *p* = 0.687) (Figure 3D).

### 3.2. ASM Effects on Vector Attraction to, and Settling on, Virus-Infected Plants

**Figure 4:**
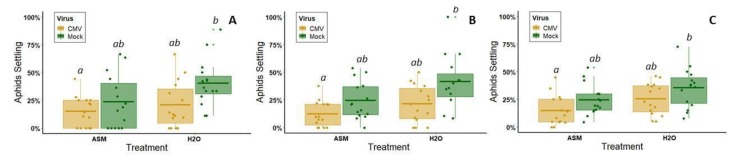
Results of alate aphid four-way choice tests between leaves from ASM+CMV, ASM only, CMV only, or non-infected control plants (*n* = 15 iterations of the assay). (**A**) Percentage of responding aphids on each of the four leaves 1 h after release. (**B**) Percentage of responding aphids on each of the four leaves 2 h after release. (**C**) Percentage of responding aphids on each of the four leaves 24 h after release. Approximately 20 alate aphids were released per test. Percentage of responding aphids was used because some insects were slower than others to enter the arena, resulting in different total numbers of insects participating at different timepoints. Lowercase letters above boxes denote treatment groups that did not have significantly different results. Dots represent individual data points. The lower and upper edges of boxes represent the first and third quartiles, with the horizontal line inside representing the median value. Whiskers extend to the highest and lowest data points within 1.5× the interquartile range. Outliers beyond this range are represented by additional semi-transparent dots.

In four-way choice tests presenting CMV-infected and non-infected plants, each with or without prior ASM treatment, there was a significant effect of both virus infection (*z* = 10.783, *df* = 1176, *p* < 0.000) and ASM treatment (*z* = 9.291, *df* = 1176, *p* < 0.000), but the interaction term was not significant (*z* = 1.58, *df* = 1176, *p* = 0.835) (*n* = 15 iterations of the assay) (Figure 4). Across all time points, alate aphids preferred leaves from untreated, non-infected plants over ASM-treated, CMV-infected plants (Tukey Test at 1 h: *p* = 0.006, 2 h: *p* < 0.000, and 24 h: *p* = 0.002) (Figure 4). Aphid preferences between non-ASM-treated plants did not differ depending on infection status (Tukey test at all time points: *p* > 0.05) (Figure 4). However, ASM treatment reduced aphid attraction and settling at each time point regardless of infection status (two-way ANOVA at 1 h: *F*(1, 56) = 4.613, *p =* 0.04, 2 h: *F*(1, 56) = 8.427, *p* = 0.005, and 24 h: *F*(1, 56) = 7.181, *p* = 0.007) (Figure 4).

**Figure 5:**
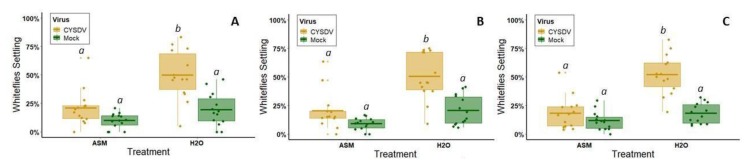
Results of whitefly four-way choice tests between leaves from ASM+CYSDV, ASM only, CYSDV only, or non-infected control plants (*n* = 14 iterations of the assay). (**A**) Percentage of responding whiteflies on each of the four leaves one hour after release. (**B**) Percentage of responding whiteflies on each of the four leaves two hours after release. (**C**) Percentage of responding whiteflies on each of the four leaves 24 h after release. Approximately 25 whiteflies were released per test. Percentage of responding whiteflies was used because some insects were slower than others to enter the arena, resulting in different total numbers of insects participating at different timepoints. Lowercase letters above boxes denote treatment groups that did not have significantly different results. Dots represent individual data points. The lower and upper edges of boxes represent the first and third quartiles, with the horizontal line inside representing the median value. Whiskers extend to the highest and lowest data points within 1.5× the interquartile range. Outliers beyond this range are represented by additional semi-transparent dots.

In four-way choice tests presenting CYSDV-infected and non-infected plants, each with or without prior ASM treatment, there was a significant effect of virus infection (*z* = −11.06, *df* = 1164, *p* < 0.000), ASM treatment (*z* = 23.08, *df* = 1164, *p* < 0.000), and their interaction (*z* = −4.19, *df* = 1164, *p* < 0.000) (*n* = 14 iterations of the assay) (Figure 5). Across all time points, significantly more whiteflies selected untreated CYSDV-infected plants relative to untreated non-infected plants and plants treated with ASM regardless of infection status (Tukey test: *p* < 0.000 for all comparisons between untreated, CYSDV-infected plants and other treatment groups) (Figure 5). ASM treatment prior to inoculation significantly reduced whiteflies’ preference for CYSDV-infected plants (Tukey test for all time points: ASM-treated CYSDV-infected vs. ASM-treated non-infected plants) (Figure 5). Our data also suggest that whiteflies appear to select preferred hosts during the first hour and remain there for at least 24 h, as we did not observe defections between hours 1 and 2. We observed that whiteflies preferred non-infected plants treated with ASM the least regardless of infection status (two-way ANOVA at 1 h: *F*(1, 52) = 21.703, *p* = 0.000; 2 h: *F*(1, 52) = 27.290, *p* = 0.000; 24 h: *F*(1, 52) = 35.57, *p* = 0.000) (Figure 5).

### 3.3. Effects of ASM Treatment on Plant Size and Productivity

**Figure 6:**
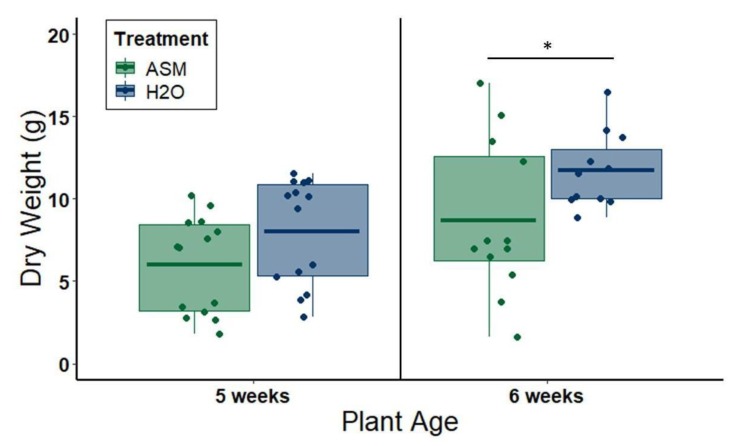
Dry weights of aboveground biomass of greenhouse-grown melon plants allowed to grow for a total of 5 or 6 weeks, respectively, following ASM applications. At 1.5 weeks old, all plants had been treated with either a foliar application of 20 mL distilled water (H2O) or 25 ppm ASM solution (ASM). Bars with asterisks denote groups between which there is a significant difference at *p* < 0.05. Dots represent individual data points. The lower and upper edges of boxes represent the first and third quartiles, with the horizontal line inside representing the median value. Whiskers extend to the highest and lowest data points within 1.5× the interquartile range. Outliers beyond this range are represented by additional semi-transparent dots.

**Figure 7:**
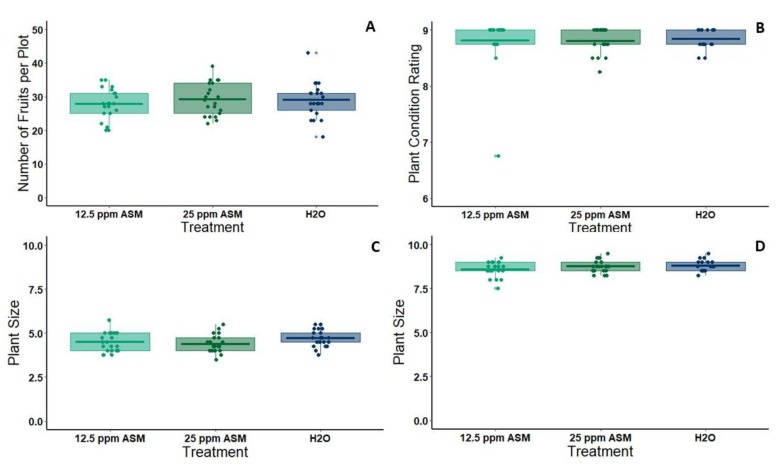
(**A**) Melon yield per plot for each of three treatment groups included in our field experiment: 12.5 ppm ASM 25 ppm ASM, and water control. (**B**) Plant condition rating (0–9 scale) per plot three weeks post-treatment for each of three treatment groups included in our field experiment: 12.5 ppm ASM 25 ppm ASM, and water control. (**C**) Plant size (1–10 scale) per plot three weeks post-treatment for each of three treatment groups included in our field experiment: 12.5 ppm ASM 25 ppm ASM, and water control. (**D**) Plant size (1–10 scale) per plot four weeks post-treatment for each of three treatment groups included in our field experiment: 12.5 ppm ASM 25 ppm ASM, and water control. For all graphs, *n* = 21 plots per treatment. There were no significant differences between treatments for any metric. Dots represent individual data points. The lower and upper edges of boxes represent the first and third quartiles, with the horizontal line inside representing the median value. Whiskers extend to the highest and lowest data points within 1.5× the interquartile range. Outliers beyond this range are represented by additional semi-transparent dots.

Non-infected melon plants treated with 25 ppm ASM in the greenhouse had slightly reduced aboveground biomass relative to non-infected plants treated with water sprays alone (3.5 weeks post-application, plants are 5 weeks old): *n* = 14 per treatment, *W* = 55, *p* = 0.051; 4.5 weeks post-application (plants are 6 weeks old): *n* = 12 per treatment, *t* = −2.26, *df* = 22, *p* = 0.034) (Figure 6). Under standard field conditions, neither one application of 12.5 ppm ASM solution nor one application of 25 ppm ASM solution affected the number of fruit produced per plot (one-way ANOVA: *df* = 2, *F* = 0.475, *p =* 0.624; Figure 7A). Additionally, there were no apparent adverse effects of ASM on melon plant condition (ANOVA: *df* = 2, *F =* 0.063, *p* = 0.939) (Figure 7B) or size (ANOVA: *df =* 2, *F* = 2.131, *p* = 0.128) (Figure 7C,D) under field conditions. 

## 4. Discussion

Viruses disrupt plant hormone signaling, induce deformities in cells, and alter the production and movement of carbohydrates, leading to detrimental changes in foliar physiology and fruit flavor [32,41,42,43,44,45,46]. For example, infection by CYSDV and the related species, *Cucurbit chlorotic yellows virus,* causes interveinal leaf chlorosis typical of viruses in the genus *Crinivirus*, and also reduces the Brix values (sweetness) of melons harvested from infected plants, rendering them unmarketable [32,42]. Further, mosaic viruses, such as CMV, induce mottling symptoms on leaves, change leaf chemistry, and cause cucurbit fruits to become misshapen, discolored, and unmarketable [24,25,43,45,47,48]. Recent studies demonstrate that the same pathologies responsible for reductions in fruit yield and quality can also exacerbate virus spread by vectors [18,46,49]. Virus-induced changes in host plant traits, such as appearance, smell, or palatability, will alter vector foraging and feeding behavior and, thereby, the probability of virus transmission [46]. Models demonstrate that virus effects on vector preferences can lead to more rapid and extensive virus spread in monocultures when they enhance vector contacts and transmission-conducive feeding behaviors [50,51,52]. However, despite the clear importance of virus symptom severity as a driver of both yield losses and virus spread, strategies for mitigating symptoms and enhancing plant tolerance to virus infection are rarely considered as components of integrated disease management.

Our results demonstrate that reductions in symptom severity and disruption of vector attraction can be achieved simultaneously through immunity modification using low doses of ASM: a commercially available mimic of the phytohormone salicylic acid. A single application of ASM successfully attenuated symptoms and delayed disease progression when administered prior to virus exposure. Furthermore, priming of the immune system induced by ASM was effective against two very distantly related viruses from different families (*Bromoviridae* and *Closteroviridae*). This suggests that ASM may be useful in scenarios where multiple, unrelated viral pathogens are endemic to a production region, or where the dominant viral pathogen varies from year to year. This is the case for our model host in this study (melons), which are infected by complexes of phylogenetically distinct viruses. Under this type of virus pressure, resistance traits generated through traditional breeding may have limited use and low economic return relative to costs associated with the breeding process and resistant material. Delaying or attenuating infection may be enough to mitigate negative effects on yield if priming is effective against a diverse suite of virus threats. 

The nature of the attenuation we observed (delay in symptoms and initial reductions in virus titer) suggests that ASM priming may act to limit both virus replication and systemic spread. A similar study exploring ASM effects on infections by tomato spotted wilt virus (genus *Orthotospovirus,* family *Tospoviridae*) in flue-cured tobacco also found evidence that ASM reduces the speed and extent of systemic virus movement [53]. A combination of limited replication and reduced systemic spread was also reported for ASM-primed tomato plants challenged with a yellowing strain of CMV [54]. For our experiments with both CYSDV and CMV, symptoms began to reappear at the same level of severity following an initial delay of approximately two weeks. For CMV, we also observed a reversal in virus titer during the final time point (3 wpi), with ASM-treated plants having higher titers than untreated plants. Thus, a dose regime that includes more than one application may be needed to prolong virus symptom attenuation and/or titer reductions. Despite limitations, the attenuation observed in our study may still be sufficient to limit virus impacts if it ultimately reduces negative effects on fruit quality or yield. Testing this will require field experiments in areas with more consistent virus pressure than the location of our field study. 

Although attenuation effects were temporary, we observed that they were sufficient to disrupt preferential vector visitation to infected hosts for one of the target pathogens (CYSDV). In the absence of ASM treatment, whitefly vectors were strongly attracted to symptomatic leaves between the 3rd and 4th week post-inoculation when symptoms rapidly appear. ASM treatment significantly reduced this preference, specifically by delaying symptom expression by two weeks. Disruption of vector attraction during this time period could have significant effects on reducing secondary spread from focal infection points. Plants typically have age-related resistance to viruses. Even susceptible genotypes of many crops are less likely to become infected beyond certain developmental stages [55]. Reducing vector attraction to infected hosts, and subsequent feeding on these hosts, provides protection for other plants in the field by reducing the probability of exposure to viruliferous vectors. Since age-related resistance varies on a weekly basis, these protective effects may significantly reduce the number of infections in the field by delaying exposure by up to two weeks. Our results also suggest that ASM may diminish exposure of non-infected plants directly by changing host quality, as our behavioral tests indicate that ASM treatment alone also slightly reduces plant palatability and attractiveness to whitefly vectors.

In contrast to results with CYSDV, for CMV, ASM pre-treatment did not significantly reduce aphid vector preferences for virus-infected plants relative to non-infected plants. In untreated melons of this variety, CMV does not enhance vector attraction, instead having slightly negative, but non-significant effects on the initial vector choice (one hour) and subsequent vector settling (2–24 h). This is somewhat contrary to previous work, which found that infection by this same isolate of CMV in cultivated squash (*Cucurbita pepo*) enhances odor cues that are attractive to multiple aphid vectors, but reduces palatability, causing vectors to visit infected plants but disperse over subsequent time periods (2–24 h) [25]. A similar attract-and-repel phenotype was reported for CMV infecting *Cucumis sativus* [26]. Based on these studies, we expected to see induction of this phenotype by CMV in *C. melo*. Our observation that a virulent genotype of CMV has no effect on aphid attraction or settling in melon therefore contributes to a growing body of evidence suggesting that virus effects on host phenotypes and vector behavior are very host-specific [18,56].

While we did not observe differences in vector attraction based on virus infection status, or virus x ASM treatment status, our results do indicate that ASM treatment alone (regardless of infection status) can reduce overall aphid vector visitation (1 h) and settling/feeding (2–24 h). As part of a separate series of experiments using electrical penetration graphing analysis (which used slightly different methods than those employed here), we confirmed that aphids do have difficulty feeding on melon plants treated with ASM; aphids spend more time searching for phloem elements and less time feeding on ASM-treated plants (Tables S2–S3). This is consistent with other, more in-depth studies of aphid resistance mechanisms in model plant systems, some of which document more rapid induction of salicylic acid-regulated defense genes in aphid-resistant genotypes following aphid attack (e.g., [57]) and reduced lifetime reproduction of aphids feeding on plants primed with ASM [58]. However, the feeding deterrence detected in our complementary EPG experiments was measured at four days post-ASM-application (vs. 3–4 weeks post-application in our free-choice settling bioassays) and was only evident with a slightly higher dose (75ppm) (Table S3). Thus, we can’t assume that the deterrence we observed in free-choice assays at 3–4 weeks post-ASM application is due to the same mechanisms, as we did not perform EPG experiments at this time. Nonetheless, we expect that both early and late aphid deterrent effects of ASM will help to reduce infection rates and virus spread, especially when considered alongside other benefits of ASM, such as reductions in virus titer.

Despite multiple benefits of ASM priming for symptom attenuation and disruption of transmission-conducive vector behaviors, we also detected slight negative effects of ASM priming on plant growth. In the greenhouse, 25 ppm ASM treatment reduced plant size, and this effect continued for one to two weeks beyond the period in which we observed symptom attenuation. These effects are consistent with prior studies documenting growth reductions for mutants overproducing salicylic acid [59]. Salicylic acid causes repression of auxin-related genes and, ultimately, inhibition of auxin responses [60]. As auxin is the main hormone regulating plant development, it is logical to presume that excessive tissue levels of salicylic acid, or mimics such as ASM, could cause phenotypes similar to auxin-deficient or auxin-insensitive mutants. However, our complementary field study suggests that under standard growing conditions, ASM-induced reductions in growth do not translate into reduced yields. This is not unusual for transplanted melons, which have also been reported to recover from phytotoxic effects of herbicide exposure at the seedling stage (including stunting) under standard agronomic conditions [61]. It is important to note that we only tested ASM on one melon variety (Gold Express), a Western Shipper-type melon that is popular in most major U.S. melon growing regions. This variety is relatively robust, with good survival and tolerance of variation in field conditions. Other varieties are more sensitive, particularly those recently bred for extended shelf life. Therefore, implementing low doses of ASM as a tool in integrated disease management of virus complexes will require additional testing under field conditions that include variation in virus pressure, cultivar choice, and agronomic practices. Despite these limitations, our results strongly suggest that plant priming is a viable option for attenuating negative effects of virus infection on plant physiology and reducing symptoms that enhance vector attraction and virus acquisition. 

## Supplementary Materials

Supplementary Materials can be found at https://www.mdpi.com/1999-4915/12/3/257/s1.
