# Supplementary material for: Priming Melon Defenses with Acibenzolar-S-methyl Attenuates Infections by Phylogenetically Distinct Viruses and Diminishes Vector Preferences for Infected Hosts
Source: Viruses. 2020 Feb 26;12(3):257. doi: 10.3390/v12030257 (PMC7150938; doi:10.3390/v12030257)
Supplement: Supplementary file 1 [file viruses-12-00257-s001.pdf]

Supplemental Material

Kenney, JR, Grandmont, M-E, Mauck, KE (2019) Priming melon defenses with acibenzolar-*S*-methyl attenuates infections by phylogenetically distinct viruses and diminishes vector preferences for infected hosts.

Table S1. Oligonucleotide primers used for qPCR analysis.

Primer Name	Sequence (5'→3')	Amplification Target	Reference
CYSDV F	CTGATGATGGGAAGGTTAGAGTGG	CYSDV	Papayiannis et al., 2010
CYSDV R	AATCTGACCTTCGGATCGGG	CYSDV	Papayiannis et al., 2010
CMV-2a F	ATGAGCTCCTTGTCGCTTTTG	CMV RNA2 ORF 2a	Feng et al., 2006
CMV-2a R	TTATTAAACGCAGGGCACCAT	CMV RNA2 ORF 2a	Feng et al., 2006
CmACT F	CCTGGTATCGCTGACCGTAT	*Cucumis melo* β-actin	Kong et al., 2014
CmACT R	TACTGAGCGATGCAAGGATG	*Cucumis melo* β-actin	Kong et al., 2014

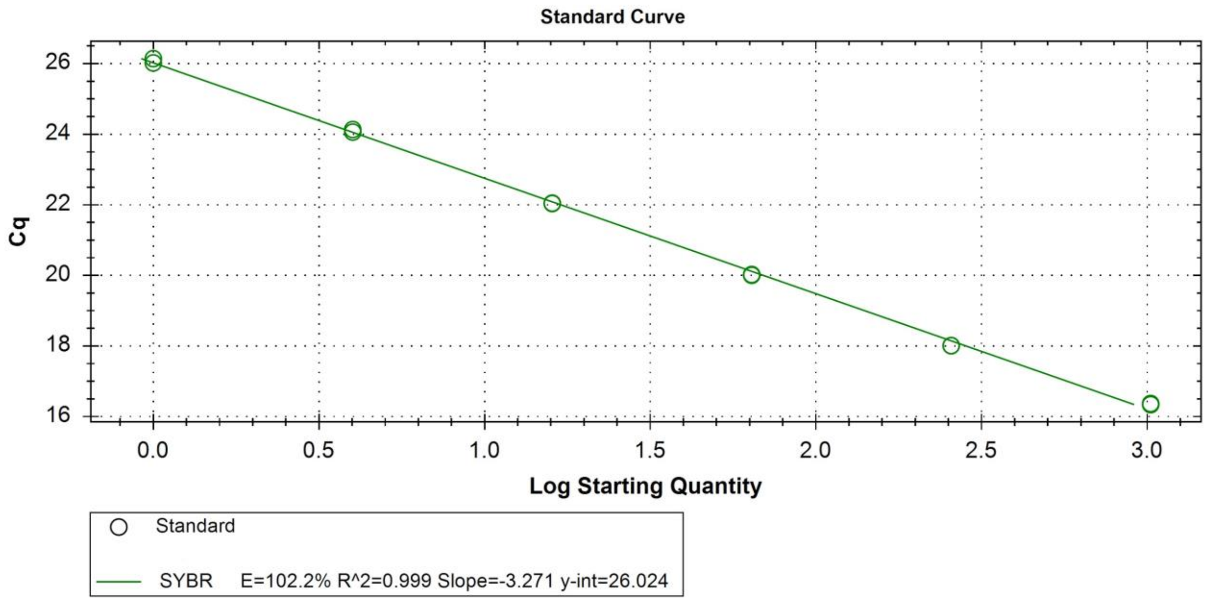

Figure S1. Standard curve and amplification efficiency (102.2%) of primerset CYSDV F and CYSDV R as calculated by CFX Manager Software (BioRad).

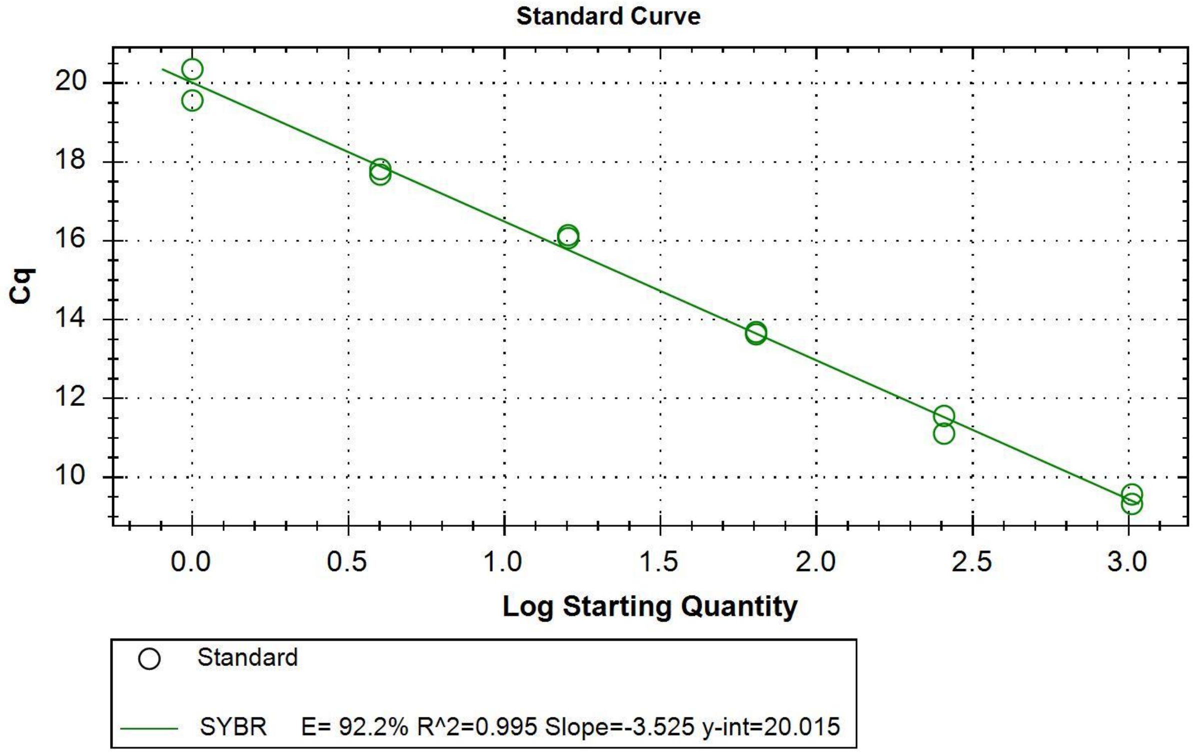

Figure S2. Standard curve and amplification efficiency (92.2%) of primerset CMV-2a F and CMV-2a R as calculated by CFX Manager Software (BioRad).

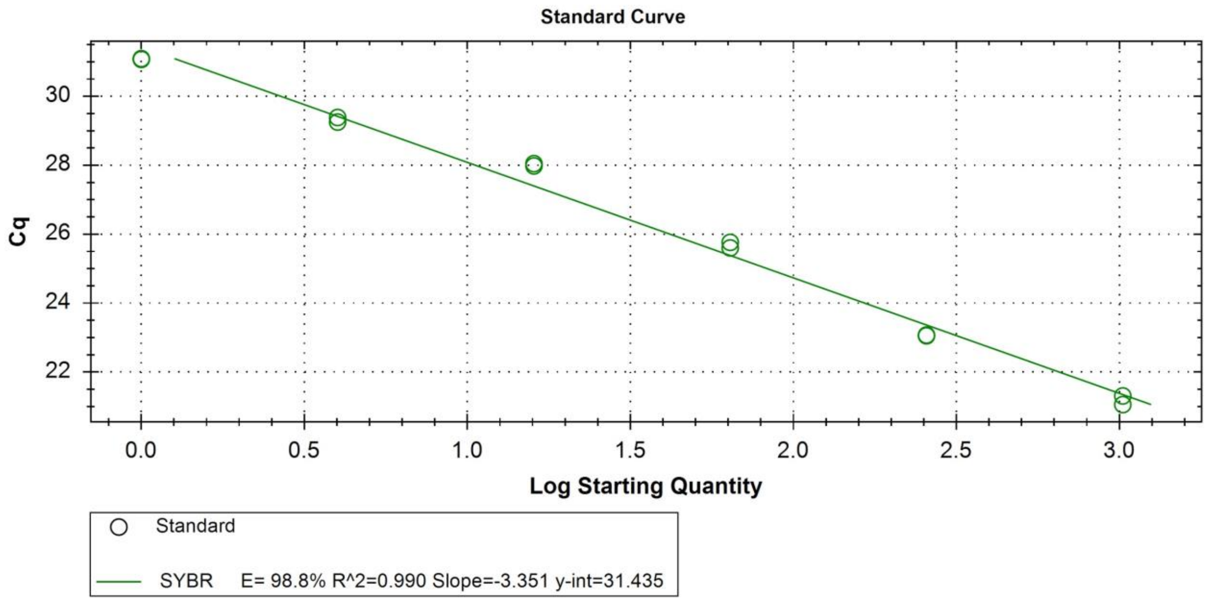

Figure S3. Standard curve and amplification efficiency (98.8%) of primerset CmACT F and CmACT R as calculated by CFX Manager Software (BioRad).

Methods for Complementary Electrical Penetration Graphing Experiments

Plants, Virus isolates, and Vectors

EPG experiments were carried out with melon, *Cucumis melo* var. Gold Express (Syngenta Seeds Inc.), germinated in seed flats in a climate-controlled growth chamber (25±1°C, 65% relative humidity) under a 16-h light/8-hr dark photoperiod. 1.5 weeks after sowing, seedlings were transplanted to 6 inch diameter x 5.75 inch tall pots (Kord Regal Standard Pots, Greenhouse Megastore) and moved to the greenhouse, where natural light and supplemental fluorescent shop lights provided a 16-h light/8-hr dark photoperiod. Plants were treated with a foliar spray that delivered 0.5mg of ASM per plant (equivalent to 25ppm) or 1.5mg of ASM per plant (equivalent to 75ppm) at the one true leaf stage, approximately 2 weeks after sowing and one to three days after transplanting and moving to the greenhouse. Control plants were treated with a foliar spray of 20 ml of distilled water.The *Aphis gossypii* colony used for this study originated from aphids collected from squash near Reedley, CA about a decade ago and reared on melon since then [1]. Colonies used here were reared on melon in the laboratory under climate-controlled conditions of 24±2°C and supplemental LED lighting providing a 16-h light/8-hr dark photoperiod.

Electrical penetration graphing (EPG) recording and analysis

Melon plants used for EPG recordings were treated with relevant ASM doses four days prior to use in experiments. For each dose level, 22-24 plants were used per treatment (ASM and Control). EPG recordings were collected using a DC-EPG system previously described by [2] and as discussed in our prior publications [3]. To create electrical circuits that included a plant and aphid individual, we tethered the insects using a 12.5 micrometer diameter gold wire attached to the pronotum with water-soluble silver conductive glue. Aphids were starved for ~30 minutes before positioning on the abaxial face of the leaf, which was immobilized and turned slightly upward to facilitate observations of insects during the experiment. Recordings were performed over four days using 12 collections per day (6 per treatment). The EPG systems were housed inside Faraday cages located in a climate controlled room (24+/- 1 degree Celsius). We used the PROBE 3.5 software (EPG Systems, www.epgsystems.eu) to acquire and analyze EPG waveforms and relevant EPG variables (naming convention based on [4]) were calculated with EPG-Calc 6.1 software [5]. We chose variables based on four different EPG waveforms described by [2,6] corresponding to: (C) stylet pathways in plant tissues except phloem and xylem; (pd) potential drops (intracellular stylet punctures); (E1) salivation in phloem elements; and (E2) passive phloem sap ingestion. The total probing time (time with stylet inserted into the plant tissue) was also considered. Mean values are given with their standard error of the mean (SEM). As aphid feeding behavior data were not normally distributed, we used Mann-Whitney U tests with alpha $P < 0.05$ for all comparisons.

Results

Table S2. Effects of 0.5mg/plant ASM application on probing and feeding behaviors of *Aphis gossypii* on melon.

EPG parameter	Control	ASM 0.5mg	Statistics
	Mean ± SEM	Mean ± SEM	
Mean number of potential drops (pd)	95.783 ± 10.345 (N = 23)	141.250 ± 20.505 (N = 24)	W = 339.5, p-value = 0.1799
Mean number of probes (Pr)	10.522 ± 1.714 (N = 23)	10.667 ± 1.336 (N = 24)	W = 300.5, p-value = 0.6075
Total duration of probe (Pr) (min)	457.002 ± 3.157 (N = 23)	450.199 ± 5.491 (N = 24)	W = 240, p-value = 0.4535
Mean number of intercellular phase (C)	11.217 ± 1.761 (N = 23)	12.250 ± 1.445 (N = 24)	W = 319.5, p-value = 0.3592
Total duration of intercellular phase (C) (min)	82.372 ± 9.305 (N = 23)	115.617 ± 16.893 (N = 24)	W = 333, p-value = 0.2316
Mean nomber of salivation phase (E1)	1.652 ± 0.305 (N = 23)	2.458 ± 0.565 (N = 23)	W = 321, p-value = 0.2837
Total duration of salivation phase (E1) (min)	1.175 ± 0.259 (N = 23)	9.177 ± 4.306 (N = 23)	W = 315.5, p-value = 0.4071
Mean number of phloem sap ingestion phase (E2)	1.565 ± 0.273 (N = 23)	2.042 ± 0.388 (N = 23)	W = 318.5, p-value = 0.2958
Total duration of phloem sap ingestion phase (E2) (min)	365.413 ± 12.574 (N = 23)	335.639 ± 16.330 (N = 23)	W = 201, p-value = 0.1134
Mean time to reach the first salivation phase (t -> E1) (min)	82.238 ± 7.606 (N = 23)	101.624 ± 19.655 (N = 23)	W = 271, p-value = 0.9244

Table S3. Effects of 1.5mg/plant ASM application on probing and feeding behaviors of *Aphis gossypii* on melon.

EPG parameter	Control	ASM 1.5mg	Statistics
	Mean ± SEM	Mean ± SEM	
Mean number of potential drops (pd)	97.636 ± 13.179 (N = 22)	175.261 ± 21.606 (N = 23)	W = 371.5, p-value = **0.006386**
Mean number of probes (Pr)	8.000 ± 1.364 (N = 22)	14.261 ± 2.295 (N = 23)	W = 368, p-value = **0.008034**
Total duration of probe (Pr) (min)	462.549 ± 3.295 (N = 22)	445.940 ± 7.312 (N = 23)	W = 158, p-value = **0.03093**
Mean number of intercellular phase (C)	10.727 ± 1.727 (N = 22)	18.696 ± 2.476 (N = 23)	W = 368, p-value = **0.008158**
Total duration of intercellular phase (C) (min)	97.502 ± 13.814 (N = 22)	160.521 ± 19.862 (N = 23)	W = 356, p-value = **0.01889**
Mean nomber of salivation phase (E1)	2.364 ± 0.434 (N = 22)	3.696 ± 0.472 (N = 23)	W = 348.5, p-value = **0.02256**
Total duration of salivation phase (E1) (min)	7.170 ± 3.313 (N = 22)	17.879 ± 4.428 (N = 23)	W = 381, p-value = **0.003138**
Mean number of phloem sap ingestion phase (E2)	1.682 ± 0.266 (N = 22)	2.043 ± 0.347 (N = 23)	W = 283.5, p-value = 0.4481
Total duration of phloem sap ingestion phase (E2) (min)	332.323 ± 19.096 (N = 22)	251.477 ± 24.739 (N = 23)	W = 148, p-value = **0.0166**
Mean time to reach the first salivation phase (t -> E1) (min)	84.429 ± 11.454 (N = 22)	98.816 ± 13.023 (N = 23)	W = 284, p-value = 0.4923

The lower dose of ASM (0.5mg per plant, corresponding to a concentration of 25ppm) did not significantly influence aphid behaviors at four days post-application. However, the larger dose (1.5mg per plant, corresponding to a concentration of 75ppm) elicited changes in several aphid behaviors. Aphids performed more potential drops (pd), also known as intracellular punctures, on 1.5mg ASM-treated plants. They also initiated more probing events (stylet withdrawals and

re-entries), more pathway phase events (C), and more salivation events (E1) (Table S2). Even though they initiated more probes, the total probing time in the plant was significantly higher on control plants. This indicates that controls may be more suitable for feeding, as aphids spent less time reinitiating probes and more time actually in the plant tissue on controls vs. 1.5mg ASM-treated plants. Reduced host quality of 1.5mg ASM-treated plants is also apparent from several other parameters measuring the durations of specific behaviors. Aphids spent significantly more time in the pathway (C) phase, engaged in longer periods of phloem salivation (E1), and spent less time ingesting phloem sap on 1.5mg ASM-treated plants relative to controls. Thus, when feeding on 1.5mg ASM-treated plants, aphids must work harder to get to the phloem and secrete more saliva into the phloem to ultimately ingest a smaller quantity of sap.

References

1. Peng, H.-C.; Walker, G.P. Sieve element occlusion provides resistance against *Aphis gossypii* in TGR-1551 melons. *Insect Sci.* **2018**.
2. Tjallingii, W.F. Electrical recording of stylet penetration activities. In *Aphids, their biology, natural enemies and control*; Elsevier Science Publishers: Amsterdam, **1988**; pp. 95–108.
3. Chesnais, Q.; Mauck, K.E. Choice of tethering material influences the magnitude and significance of treatment effects in whitefly electrical penetration graph recordings. *J. Insect Behav.* **2018**, *31*, 656–671.
4. Ebert, T.A.; Backus, E.A.; Cid, M.; Fereres, A.; Rogers, M.E. A new SAS program for behavioral analysis of electrical penetration graph data. *Comput. Electron. Agric.* **2015**, *116*, 80–87.
5. Giordanengo, P. EPG-Calc: a PHP-based script to calculate electrical penetration graph (EPG) parameters. *Arthropod Plant Interact.* **2014**, *8*, 163–169.
6. Tjallingii, W.F.; Esch, T.H.H. Fine structure of aphid stylet routes in plant tissues in correlation with EPG signals. *Physiol. Entomol.* **1993**, *18*, 317–328.
